# Wasted Biomaterials from Crustaceans as a Compliant Natural Product Regarding Microbiological, Antibacterial Properties and Heavy Metal Content for Reuse in Blue Bioeconomy: A Preliminary Study

**DOI:** 10.3390/ma14164558

**Published:** 2021-08-13

**Authors:** Fran Nekvapil, Iolanda-Veronica Ganea, Alexandra Ciorîță, Razvan Hirian, Lovro Ogresta, Branko Glamuzina, Carmen Roba, Simona Cintă Pinzaru

**Affiliations:** 1 Ioan Ursu Institute, Babeș-Bolyai University, 1 Kogălniceanu, 400084 Cluj-Napoca, Romania; fran.nekvapil@ubbcluj.ro (F.N.); razvan.hirian@ubbcluj.ro (R.H.); lovroogresta4@gmail.com (L.O.); 2 Physics of Nanostructured Systems Department, National Institute for Research and Development of Isotopic and Molecular Technologies, 67-103 Donat, 400293 Cluj-Napoca, Romania; iolanda.ganea@itim-cj.ro; 3 RDI Laboratory of Applied Raman Spectroscopy, RDI Institute of Applied Natural Sciences (IRDI-ANS), Babeş-Bolyai University, Fântânele 42, 400293 Cluj-Napoca, Romania; 4 Faculty of Environmental Science and Engineering, Babeș-Bolyai University, 30 Fântânele, 400294 Cluj-Napoca, Romania; carmen.roba@ubbcluj.ro; 5 Faculty of Biology and Geology, Babeș-Bolyai University, 44 Republicii St., 400015 Cluj-Napoca, Romania; alexandra.ciorita@itim-cj.ro; 6 Integrated Electron Microscopy Laboratory, National Institute for Research and Development of Isotopic and Molecular Technologies, 67-103 Donat St., 400293 Cluj-Napoca, Romania; 7 Department of Applied Marine Ecology, University of Dubrovnik, Ćira Carića 4, 20000 Dubrovnik, Croatia; branko.glamuzina@unidu.hr

**Keywords:** crab shells waste, biofertilizer, microbial content, heavy metals, environmental regulations

## Abstract

The compliance of crab shells traditionally used as a complex natural product for agricultural soil amendment with modern biofertilizers’ quality and safety requirements was investigated. Shells waste from the Blue crab, *Callinectes sapidus* and the Green crab, *Carcinus aestuarii* were tested for macronutrients, heavy metals, bacteria content, and antimicrobial properties. Such information is crucial for further utilization of the biogenic powders for any composite formulation in added-value by-products. The calcium carbonate-rich hard tissue yield was 52.13% ± 0.015 (mean ± S.D.) and 64.71% ± 0.144 from the blue and green crabs, respectively. The contents of Pb, Ni, Zn, Cr (VI), and Cu were several orders of magnitude below the prescribed limit by EU biofertilizer legislation, with Fe, Mn (not prescribed), and As being the most abundant. The content of As and Cd from the material considered here was within limits. The shells contain no colony-forming units of *Salmonella* spp. and compliant levels of *Escherichia coli*; moreover, the shell micro-powder showed dose-dependent growth inhibition of *Pseudomonas aeruginosa* and *Staphylococcus aureus*. In summary, the waste crab shells present a complex natural product as plant biofertilizer following the circular economy concepts.

## 1. Introduction

In an era driven by global environmental changes, sustainable agricultural practices using molecular and complex natural products have the potential to become key alternatives towards the transition to a green circular economy [1,2]. For decades, it has been known that marine organisms, especially the crustacean shells, are a reservoir of still insufficiently exploited or resources [1,2]. Crab shells are a known source of chitin [1]; however, the utilization of the shell biocomposite as a whole has only recently been a subject of deeper technological investigations [3,4,5,6]. The safe and sustainable use of crabs in agrotechnology requires a knowledge-based approach and insights into which chemical or microbiological parameters are critical to avoid potential environmental pollution or crop under-performance.

Although the benefits and drawbacks of the application of natural calcium carbonate [7,8] and ground leftover crab shells to agricultural crops are already recorded in the current literature [9,10,11], update is needed on the compliance of these nature-derived materials with the requirements for employing them into new biofertilizer formulations. This is particularly true particularly for soils from the harvesting and/or processing areas, to avoid environmental pollution and waste management costs.

The “EU Fertilizer Regulation” [12] is used here as a framework piece of legislation introducing biofertilizer categories, and quality and safety standards. It also recognizes the necessity of reusing organic matter as biofertilizers and biostimulants, representing promising sustainable substitutes for synthetic products or plant growth regulators [13].

The color-specific porosity of the blue crab (species *Callinectes sapidus*, native to North America, invasive in the Mediterranean) and the green crab (species *Carcinus aestuarii,* native to the Mediterranean) shells has been recently comprehensively investigated by multiple techniques [4,5]. Spectroscopy, diffraction, and thermogravimetry techniques showed that the bulk crab claw shells consist of Mg-calcite (75–85% by weight) mineralized on a chitin-rich organic fibrous scaffold (14–19% by weight), and additionally contain carotenoid pigments and carotenoproteins [4,5]. This unique combination of bioactive organic compounds, carbonate minerals, and nanoporosity makes crab shells an attractive complex natural product for various niches of agricultural applications or other smart biocomposites for wider applicability, as previously described [4]. Specifically related to agriculture, Panhwar et al. [7] argued that the amendment of rice farming soil with ground magnesian limestone in combination with an organic fertilizer acted to elevate the soil pH, and exchangeable Ca and Mg nutrients, which in turn enhanced the plant growth. This observation [7] opens the avenue for the use of crab shells for fertilizing purposes.

Here, based on our previous reported structural and biochemical data on crab shells [4,5], we investigated the microbiology properties of the shell powder and its potential heavy metal accumulation. Such information is crucial for further utilization of the respective biogenic powders for any added-value by-products. Additionally, Raman spectroscopy of unpigmented (white) shell fragments has been employed here for a fast assessment of the crystallinity and chemical composition of the material. The long-term aim is to highlight the immediate potential of recycling crustacean shell waste, which is currently being landfilled at a large scale, as a secondary raw material for novel fertilizing products following the principles of the Blue bioeconomy and the circular economy.

## 2. Materials and Methods

### 2.1. The Samples and the Powdering Procedure

Shells of the two crab species, the Atlantic blue crab (*Callinectes sapidus*) and the Mediterranean green crab (*Carcinus aestuarii*) [4,5], were considered here. Crab specimens are caught periodically for the purpose of collaborative experiments between the Babeș-Bolyai University and the University of Dubrovnik, in Parilla lagoon (Neretva river delta), which is historically an agricultural and industrial region of Croatia and Bosnia and Herzegovina, respectively.

This study did not require formal institutional ethical approval; however, specimens were handled according to the best available animal welfare in science practices and institutional ethical recommendations [4,5,6].

We treated different shell anatomical parts separately due to previous findings regarding their distinct porosity [4,5]. Thus, previously studied shell categories (blue, red, white shells from *C. sapidus* and green shells from *C. aestuarii*) were complemented here by the carapaces and a domestically cooked shell batch (considered as food waste) where relevant.

To estimate the amount of biomineral phase obtained from crabs, three blue crab and six green crab specimens were dissected and cleaned from the soft tissue. All shell categories were selectively weighted with 0.1 g precision.

For microbiological testing of real-world crab shell waste, a 10 kg stock of blue crab and the green crab shells resulting from restaurant preparation was randomly subsampled. A part of this stock of waste shells was washed from residual organic material by rinsing for a minute in running warm tap water (40 °C) and termed “rinsed shells.” The remaining shells with organic material residues were termed “waste shells.”

For preparing powders, three individuals of *C. sapidus* and six *C. aestuarii* specimens obtained during a 1-year period were employed. For milling into micropowder, shell fragments from each category were air-dried to preserve the potential coliform bacteria community for later microbiology tests. Subsequently, shells were manually pre-grinded in an agate mortar and then finely milled in a Fritsch Pulverisette 4 planetary ball mill (Fritsch, Oberstein, Germany). Two hardened steel bowls were used for milling, depending on the amount of material to be milled: the 80 mL bowls (1 to 12 g of shells) and 500 mL bowls (35 to 50 g of shells). Steel balls (10 mm diameter; 23 balls per bowl) were used. Shells were milled in single 5-min cycles with the disc/bowls rotation speed of Ω/ω = 333/900, as Nekvapil et al. [4] described in the previously reported thermal treatment experiment. All milled samples were sieved through a 100-μm steel mesh. The particles that did not pass the sieve were discarded (around 2% of the total mass).

### 2.2. Raman Spectroscopy

Raman spectra were acquired from the unpigmented, white claw shell areas of ten *C. sapidus* specimens collected over five years to track the bulk shell mineral composition. The Renishaw inVia Reflex confocal Raman microscope (Renishaw, Wotton-under-Edge, UK) was used, and the spectra were acquired using the 532 nm excitation (Cobolt diode-pumped solid state, DPSS laser; Cobolt AB, Solna, Sweden) and the 20× magnification objective (NA 0.35). The spectral resolution was 0.5 cm^−1^, with the analysis area diameter on the sample surface of 1.85 μm. Shell was cut into 5 × 5 mm pieces which are manageable on the XYZ electronically controlled microscope stage.

### 2.3. Heavy Metals Analysis

Samples of 0.4 g blue crab claw dactyls, blue claw shells, and green crab claw shells were prepared for the heavy metal content analysis by applying a prior microwave-assisted digestion procedure consisting of 5 mL HNO_3_, 5 mL H_2_O_2_, and 3 mL H_2_O. The microwave programs included four heating steps with a 2 min ramp: the procedure involved heating for 5 min at 145 °C, 5 min at 170 °C, 18 min at 180 °C, and 3 min at 75 °C. After cooling, the samples were transferred to volumetric flasks and diluted to a final volume of 25 mL with 0.2% HNO_3_. Analytical blanks were also prepared using the same procedure. Samples were further analyzed with a ZEEnit 700 Atomic Absorption Spectrometer (AAS) (Analytik Jena, Jena, Germany) equipped with single-element hollow cathode lamps, an air-acetylene flame, and a graphite furnace.

Arsenic (As) content was determined by inductively coupled plasma mass spectrometry (ICP-MS) using an ELAN DRC II spectrometer (Perkin Elmer, Waltham, MA, USA) and aqua regia (3/1 HCl/HNO_3_) extraction.

Hexavalent chromium (Cr VI) was determined from 1:10 leachate by the spectrophotometric method according to SR ISO 11083: 1994 [14] using 1,5-diphenylcarbazide with UV-VIS spectrophotometer (Perkin Elmer, Waltham, MA, USA). The detection limit was 0.1 mg kg^−1^.

### 2.4. Microbiological Analysis

#### 2.4.1. Bacterial Cultures

To determine if the prepared crab shell powders are suitable for their use as biofertilizers, microbiological analyses were performed according to the legal requirements [12]. Selective agar media for *Salmonella* spp., *Enterococcus* spp., and *E. coli* were prepared. The crab shell powders (100 mg) were dispersed in 1 mL of phosphate buffered saline (PBS) 0.1 M, and serial suspensions of progressively lower powder mass fraction (up to 10^−10^) were performed. Hence, 1 mL of each resulting suspension was dispersed on the agar plates. The media was left to infiltrate with the suspension for 30 min, after which the plates were incubated for 24 h at 35 °C. After this time elapsed, the colony-forming units (CFU) were counted. Each experiment was conducted three times at each dispersion rate.

#### 2.4.2. Antibacterial Properties

To determine the antibacterial effects of the crab shell powders, four bacterial strains were tested through the microdilution method, according to EUCAST protocols [15,16]. As Gram-negative models, *E. coli* (ATCC: 25922) and *Pseudomonas aeruginosa* (ATCC: 27853) were used, and as Gram-positive models, *Enterococcus faecalis* (ATCC: 29212) and *Staphylococcus aureus* (ATCC: 25923), cultured overnight in Mueller–Hinton (MH) agar, at 35 °C were used. The 24 h cultures were adjusted to 0.5 McFarland turbidity and cultured in 96 well plates with the powders in different concentrations. The crab shell powders were dispersed in PBS and brought to a 10 mg mL^−1^ concentration. Each plate had the first column as untreated control, with media and bacterial suspension, the last column as the positive control (ciprofloxacin at 10 µg mL^−1^ final concentration), negative controls with media only, vehicle controls with media and shell powders, and 10 columns with decreasing concentrations of crab shell powder (range from 0.008 to 4.5 mg mL^−1^), all adjusted to a final volume of 110 µL. The plates were then incubated for 18–20 h at 35 °C, after which the absorbance was read at 600 nm (Epoch plate reader; BioTek, Bad Friedrichshal, Germany). The percentage of inhibition was calculated based on the observed absorbances (the absorbance divided by the untreated control and times 100), and the minimum inhibitory concentration (MIC) was estimated. Each experiment had six replicas, and the mean was calculated from at least three independent results.

## 3. Results

### 3.1. Crab Shell Powdering Technical Aspects

**Figure 1:**
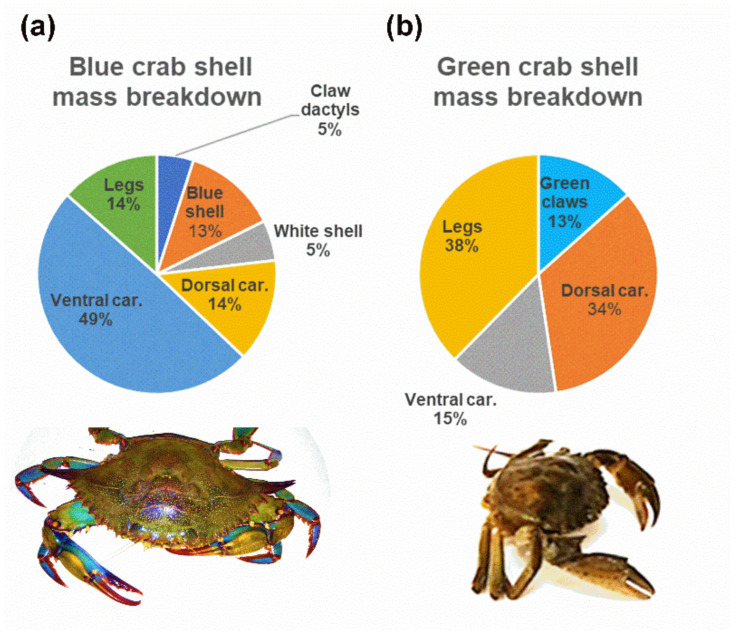
Weight fractions of particular anatomical shell parts of the two crustacean species: (**a**) the Atlantic blue crab (*Callinectes sapidus*) and (**b**) the Mediterranean green crab (*Carcinus aestuarii*). Car = carapace.

The total mineral (shell) mass fraction of the blue crab, *C. sapidus* amounts 52.13% ± 0.015 (mean ± standard deviation), while the fraction for the green crab, *C. aestuarii*, is the fraction higher, 64.71% ± 0.144. The shell mass breakdown by shell types is presented in Figure 1.

The size of powder particles is lowered below 100 μm after only 5 min of milling, as determined by dynamic light scattering (DLS; Figure S1). Interestingly, the blue-colored shells and the claw dactyls also yielded a nano-sized particle fraction of about 20% by number, with a size of less than 500 nm (Figure S1, red highlight). This is presumably related to the appearance of fines related to the shell mineral structure, with the blue shells having the lowest and the dactyls having higher CaCO_3_ crystallinity [5].

### 3.2. Raman Spectroscopy Tracking of Mineral Composition

**Figure 2:**
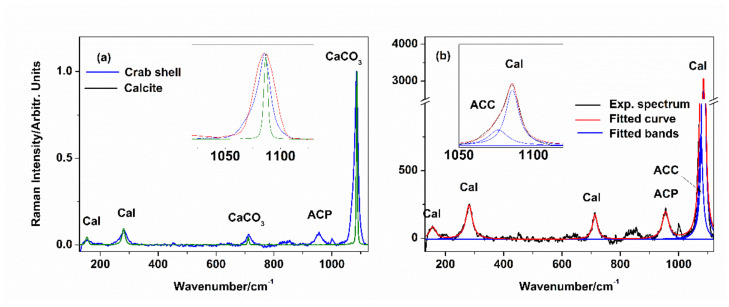
(**a**) Comparative Raman spectra of the bulk blue crab *Callinectes sapidus* unpigmented shell, and the FT-Raman spectrum of synthetic calcite; insert shows the zoom of 1030 to 1130 cm^−1^ range containing the CaCO_3_ v_1_(CO_3_^2−^) band. (**b**) Averaged Raman spectrum (29 spectra, background subtracted) acquired from the white shell areas of the Atlantic blue crab, *C. sapidus*, and fitted with Lorentzian profiles. Insert shows the zoom of the v_1_(CO_3_^2−^) band. Band labels of minerals: Cal = calcite; ACC = amorphous calcium carbonate; CaCO_3_ = shared bands of calcite and ACC; ACP = amorphous calcium phosphate Excitation: crab shell 532 nm, synthetic calcite 1064 nm.

The Raman spectra acquired from the unpigmented (white) crab shell and the synthetic CaCO_3_ are shown comparatively in Figure 2a. Crab shell spectra featured bands assigned to calcite [17] (crystalline CaCO_3_) at 156, 281, 712, and 1085 cm^−1^, and a broadened band attributable to amorphous CaCO_3_ (ACC) at 1076 cm^−1^. Lastly, the band at 955 cm^−1^ is assigned to amorphous calcium phosphate (Figure 2b). Although chitin Raman bands have been reported previously in equivalent shell fragments [4,5], the laser excitation line at 532 nm provided a high fluorescence background that covers the chitin peaks. The area ratios of calcite v_1_(CO_3_^2−^) at 1085 cm^−1^ and the corresponding band assigned to ACC, R = A_(1085)_/A_(1076)_ was 2.44, close to the 2.82 reported by Nekvapil et al. [5] and 2.3 reported by Katsikini [18]. Following the relationships of band positions and widths to the degree of Mg substitution reported previously for calcite [19] and ACC [20], the crab shells considered here would contain less than 5% Mg by weight.

### 3.3. Heavy Metals in Crab Shells

**Figure 3:**
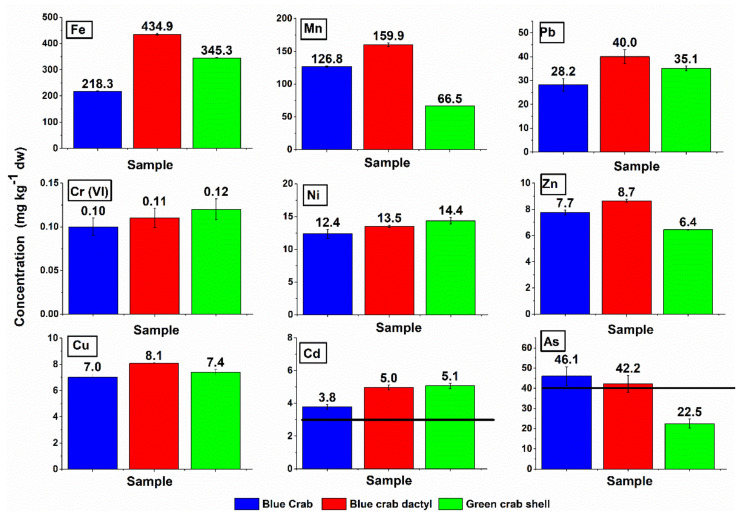
Heavy metal contents (mg kg^−1^ dw) determined in the shells of the blue crab (*Callinectes sapidus*) and the green crab (*Carcinus aestuarii*) of eastern Adriatic Sea origin, near an industrial and agricultural zone (Neretva river delta, Croatia). Error bars represent R.S.D. (*n* = 3). Black horizontal line: prescribed maximum permissible level of metal for inorganic macronutrient fertilizers [12].

Fe, Mn, and As were the most abundant metal ions determined in the studied crab shell parts, while Zn, Cu, and Cd were present in lower concentrations (Figure 3). Accumulation of As and Cd exceeding the level of the current regulation was observed in the crab shells. The sediment arsenic content in the current crabs’ collection site was recently reported between 18.7 and 47.2 mg kg^−1^ [21]; since crabs are bottom-dwelling animals, the relatively high As content in crab shells of 22.5 to 46.1 mg kg^−1^ was not so surprising. The wind-borne pollution can explain shell cadmium content from a bauxite terminal in the vicinity of the collection site.

### 3.4. Microbiology

At 24 h post-incubation, three colony-forming units (CFU mL^−1^) were detected on the plate incubated with rinsed shells (rinsed food waste stock) (Table 1). In contrast, the plate incubated with the waste shells had 81 CFU mL^−1^ of two different types of bacteria (Figure S2). Three colony-forming units were observed on one plate cultivated with the green shell powder dispersion, identified morphologically as *Enterococcus spp*., as well. Some of the samples were left for an additional 24 h, and subsequently, one CFU mL^−1^ assigned to *E. coli* was observed on one of the other Petri dishes.

**Figure 4:**
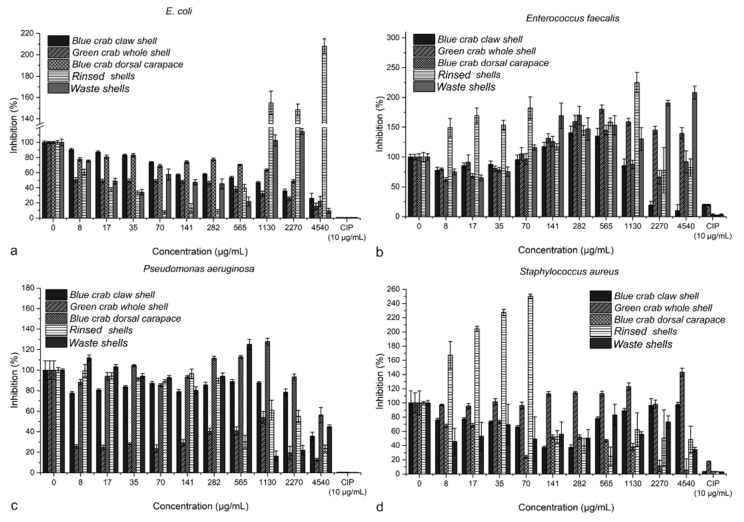
The antibacterial effects of crab shell powders against *Escherichia coli* (**a**), *Enterococcus faecalis* (**b**), *Pseudomonas aeruginosa* (**c**), and *Staphylococcus aureus* (**d**), assessed through the microdilution method. CIP = ciprofloxacin.

The antibacterial effect of the shell powders was assessed through the microdilution method, and the concentration at which 50% of the bacteria are inhibited (MIC) was calculated (Table 2). The bacteria reaction was dose-dependent in most cases (Figure 4). However, not all strains had a negative correlation in a dose-dependent manner. For *E. faecalis*, the absorbance increased with the increasing shell powder concentration when treated with waste shells; however, the high amount of CFU mL^−1^ (40 CFU mL^−1^) of *Enterococcus spp*. detected in the respective powder in the above test validates the former claim. In *E. coli*, both the powders produced from rinsed and waste shells exhibited a dose-dependent proliferative effect against this strain.

## 4. Discussion

The potential and importance of recycling wasted biological material into innovative fertilizers in the spirit of the circular economy and sustainable development instead of landfilling was recognized by the leading legislative bodies, such as the European Commission [12]. However, the recycling and the final characteristics of such by-products and fertilizer complexes have to be regulated to avoid adverse effects on human wellbeing and the environment and inform and protect the interests of end-users [12]. The Fertilizer Regulation [12] introduced by the European Commission aimed to set the framework of acceptable biofertilizer categories and characteristics and will be used herein as a reference guide.

Our previous investigations using the semiquantitative energy-dispersive x-ray spectroscopy (EDX) data from crab shells showed only the main biomineral constitutive elements such as Ca, Mg, P, C, and O, with trace amounts of Na, K, S, and Cl. Al was occasionally detected; however, no other heavy metal signal was recorded [5]. Thus, we conclude the accidental accumulation of heavy metals related to the heavy metals pollution of the crabs’ habitat. An important consideration is that the shell contamination with particular metals strongly depends on the type of local environmental pollution from industrial effluent [22,23]. Similar, or slightly lower range of Fe, Cu, Pb, and Cd content than in the present study, was reported by Abdel-Salam [24] in the shell of several crab species, who suggested that the shells of respective species originating from Egyptian and Saudi Arabia coasts are suitable for use as mineral supplements for animal feed.

Our study indicates that the exceeding crab shell content of As and Cd, reported here at 22.5 to 46.1 and 3.78 to 5.07 mg kg^−1^, is a critical parameter for further processing and indicates that heavy metals analysis should be conducted on shell material when industrial pollution is suspected. This means that the crab shells like these considered here would have to be combined with another As- and Cd-free fertilizing material to reduce the content of the contaminants per unit of weight. Nevertheless, the content of other contaminants, such as Pb, Ni, Zn, Cu, and Cr (VI), is commonly 1 to 3 orders of magnitude below the legal threshold, as also evidenced by Abdel-Salam [24], still making the crab shells an attractive naturally sourced composite material for agricultural applications.

No *Salmonella spp*. CFU was detected, and the content of enteric bacteria was well below the prescribed limit [12] even in unrinsed waste shells. Because no or very few colonies forming units were in general observed on the cultured plates, the antibacterial properties were tested. The best inhibitory effect was observed for green shell powder against *P. aeruginosa* (3 µg mL^−1^) and *E. coli* (410 µg mL^−1^), followed by the blue crab dorsal carapace against *S. aureus* (760 µg mL^−1^); this is linked to the content of biopolymer chitin, which was previously detected in native crustacean shells by spectroscopic and x-ray diffraction methods [4,5,6]. Chitosan is a deacetylated form of chitin, the pharmacological potential of which is well reported in the literature [25,26]. Moreover, a moderate antibacterial (and anti-fungal) capacity of the blue crab and crab and shrimp shell had been previously reported [27,28]; hence the micropowder produced from wasted crab shells is suitable for all fertilizing product categories from the microbiological point of view. The beneficial effect of chitin on boosting the plant resistance to pathogens [29] additionally opens avenues for investigation of biostimulative effects of the shell powder.

Locally abundant populations of the blue crab were reported both in its native range and the invaded areas [30,31,32]. Given the continuous increase in crustacean fishery captures worldwide [33,34], the opportunity for exploitation of its abundant wasted shells appears attractive for useful by-products. To ensure a stable crab population, and in turn, shell supply, crab migration routes and spawning grounds have to be managed properly [35]. However, this kind of management is difficult in the invaded areas, e.g., the Mediterranean Sea, where blue crab populations directly conflict with traditional aquaculture facilities, e.g., preying on shellfish farms [36]. A SWOT analysis of the blue crab management as a seafood resource and as an invasive species in the South European waters was recently proposed by Mancinelli et al. [36]. The latter authors acknowledged the lack of in-depth studies of local blue crab population dynamics as a weakness and the waste shell material supply as an opportunity. The present study expands on the latter point with concrete structural and chemical data. Nevertheless, local risks to economic activities (e.g., shellfish farming) have to be considered to promote blue crabs as a multi-purpose sustainable commodity (crab meat and shell supply) [36].

## 5. Conclusions

This study showed that the shells of the blue and green crab could be considered legally compliant fertilizing material regarding their bulk composition and microbiological content [12]. Particular attention should be paid to the heavy metal contents of the crab shells, especially Cd and As, where industrial pollution is known in the crab collection area. This may be the critical parameter for the utilization of shells. Additional contaminants tracking will be necessary to prospect the suitability of waste crab shells for other emerging applications, such as medicine and advanced composites science.

## Figures and Tables

**Table 1:** Microbiological assessment of the rinsed shell powders, according to the requirements of the European Regulation on making available on the market of EU fertilizing products [12]. CFU = colony forming units.

Bacterial Strain	Sampling			Limit
	*n*	c	CFU/mL	
*Salmonella spp*.	1	0	0	Absence
*E. coli*	1	1	3	100 CFU
*Enterococcus spp*.	1	1	0	100 CFU

*n* = number of replicas for each sample; c = total number of samples where the bacterial strains were detected; CFU/mL = colony forming units detected in 1 mL of diluted powder of 100 mg mL^−1^ concentration.

**Table 2:** The minimal inhibitory concentration (MIC) of crab shell powder determined for each tested bacterial strain.

Powder		MIC (mg mL^−1^)			
	Bacterial Strain	*E. coli*	*P. aeruginosa*	*E. faecalis*	*S. aureus*
Blue crab claw shell		1.92	2.92	1.57	2.56
Green crab (entire shell)		0.41	0.003	6.96	6.9
Blue crab dorsal carapace		2.3	7.17	1.41	0.76
Rinsed shells		-	2.43	6.7	3.09
Waste shells		-	2.64	-	3.25

## Data Availability

The data that support the findings of this study are available within the manuscript and its Supplementary Materials. Additional information can be provided from the corresponding author upon reasonable request.

## Supplementary Materials

The following are available online at https://www.mdpi.com/article/10.3390/ma14164558/s1, Figure S1: Results of DLS particle size distribution measurements of selected shell parts. The nano-sized fraction of the blue and red shells is indicated by red ovals. Figure S2: Plates with Enterococcus spp (small colonies) and *E. coli* (large colonies). Cultures in the presence of powdered crab shells as indicated. Yellow arrows point to the CFU (colony forming units).
