# Supplementary material for: Wasted Biomaterials from Crustaceans as a Compliant Natural Product Regarding Microbiological, Antibacterial Properties and Heavy Metal Content for Reuse in Blue Bioeconomy: A Preliminary Study
Source: Materials (Basel). 2021 Aug 13;14(16):4558. doi: 10.3390/ma14164558 (PMC8399662; doi:10.3390/ma14164558)
Supplement: Supplementary file 1 [file materials-14-04558-s001.zip › materials-1278992-supplementary.pdf]

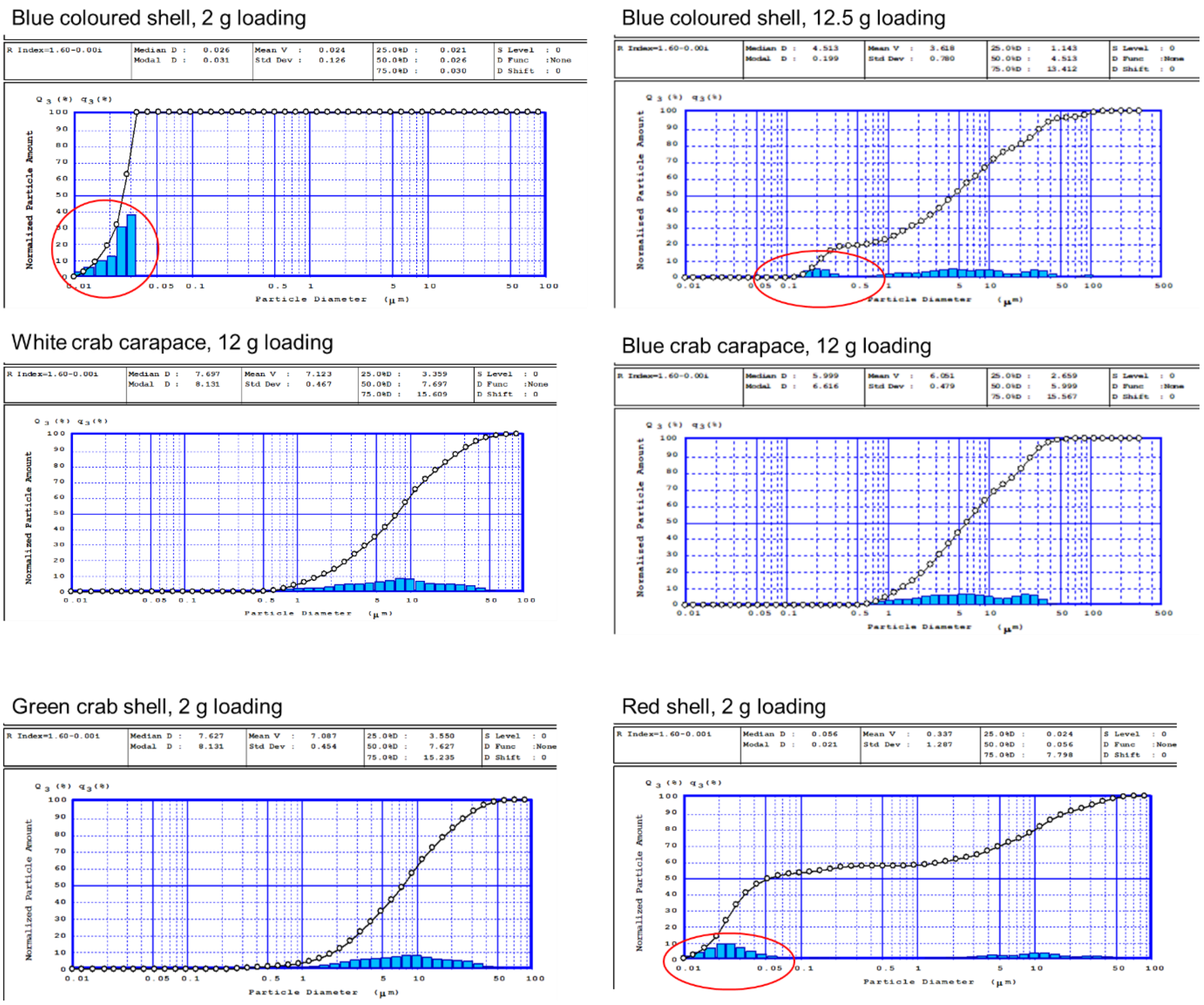

Figure S1. Results of DLS particle size distribution measurements of selected shell parts. The nano-sized fraction of the blue and red shells are indicated by red ovals.

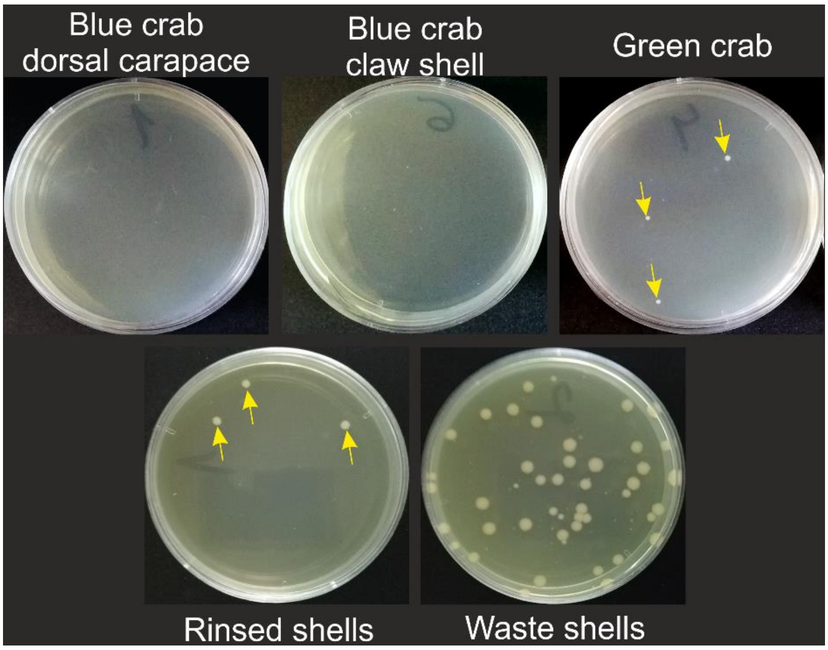

Figure S2. Plates with *Enterococcus* spp (small colonies) and *E. coli* (large colonies). Cultures in the presence of powdered crab shells as indicated. Yellow arrows point the CFU (colony forming units).
